# Risk of Mortality and Readmission among Patients with Pelvic Fracture and Urinary Tract Infection: A Population-Based Cohort Study

**DOI:** 10.3390/ijerph18094868

**Published:** 2021-05-03

**Authors:** Ying-Cheng Chen, Cheng-Hsun Chuang, Ming-Hong Hsieh, Han-Wei Yeh, Shun-Fa Yang, Chiao-Wen Lin, Ying-Tung Yeh, Jing-Yang Huang, Pei-Lun Liao, Chi-Ho Chan, Chao-Bin Yeh

**Affiliations:** 1 Institute of Medicine, Chung Shan Medical University, Taichung 402, Taiwan; chenyc66@gmail.com (Y.-C.C.); skdef37372@hotmail.com.tw (C.-H.C.); ysf@csmu.edu.tw (S.-F.Y.); wchinyang@gmail.com (J.-Y.H.); 2 Department of Surgery, Changhua Christian Hospital, Changhua 500, Taiwan; 3 School of Medicine, Chung Shan Medical University, Taichung 402, Taiwan; mhhpsy@hotmail.com; 4 Department of Emergency Medicine, Chung Shan Medical University Hospital, Taichung 402, Taiwan; 5 Department of Psychiatry, Chung Shan Medical University Hospital, Taichung 402, Taiwan; 6 School of Medicine, Chang Gung University, Taoyuan 333, Taiwan; george66889@gmail.com; 7 Department of Medical Research, Chung Shan Medical University Hospital, Taichung 402, Taiwan; liaopeilun0410@gmail.com; 8 Institute of Oral Sciences, Chung Shan Medical University, Taichung 402, Taiwan; cwlin@csmu.edu.tw; 9 Graduate School of Dentistry, School of Dentistry, Chung Shan Medical University, Taichung 402, Taiwan; yehtungtung@hotmail.com; 10 Department of Dentistry, Chung Shan Medical University Hospital, Taichung 402, Taiwan; 11 Department of Microbiology and Immunology, Chung Shan Medical University, Taichung 402, Taiwan

**Keywords:** mortality, re-admissions, urinary tract infections, pelvic fracture

## Abstract

Patients with pelvic fractures could encounter various complications during or after treatments. This cohort study investigated the risk of mortality and readmissions in patients with pelvic fractures, with or without urinary tract infections (UTIs), within 30 days following the pelvic fractures. This retrospective cohort study examined claim records from the Longitudinal Health Insurance Database 2000 (LHID2000). We selected patients hospitalized with pelvic fractures between 1997 and 2013 for study. Patients who had index data before 2000 or after 2010 (*n = *963), who died before the index date (*n = *64), who were aged <18 years (*n = *94), or who had a pelvic injury (*n = *31) were excluded. In total, the study cohort comprised 1623 adult patients; 115 had UTIs, and 1508 patients without UTIs were used as a comparison cohort. Multivariate analysis with a multiple Cox regression model and Kaplan–Meier survival analysis were performed to analyze the data. Our results showed that the 1-year mortality rate (adjusted hazard ratio [HR]: 2.32; 95% CI: 1.25–4.29) and readmission rate (adjusted HR: 1.72; 95% CI: 1.26–3.34) of the UTI group were significantly higher than those of the non-UTI group. Moreover, the Kaplan–Meier curve for the 1-year follow-up indicated that the UTI group had a higher cumulative risk of both mortality and hospital readmission compared with the non-UTI group. In conclusion, among patients with pelvic fracture, patients with UTI were associated with increased risks of mortality and readmission. Physicians must pay more attention to such patients to prevent UTIs among patients with pelvic fractures during hospitalization and conduct a follow-up after discharge within at least 1 year.

## 1. Introduction

Pelvic fractures can occur due to low-to-high energy compression and trauma. The mortality rate for severe cases of pelvic fractures could be is >40% [1]. However, patient mortality is not always caused directly by the fractures themselves; major organ injuries, hemodynamic instability, and infection are possible causes [1,2]. Unstable pelvic ring injuries and internal bleeding, especially those caused by arterial mass hemorrhage, considerably increase mortality [3,4]. Therefore, controlling serious hemorrhages, stabilizing fractures, and avoiding urinary tract injuries are interventions critical to decreasing associated mortality rates [5,6].

Certain complications can occur in patients with pelvic fractures, including pseudoaneurysms, renal failure, soft-tissue necrosis/infections, and anaphylactic reactions, especially when the approach of pelvic angiography with transcatheter arterial embolization is used to control pelvic arterial hemorrhage [7]. Pelvic ring disruption could also cause injuries to neurovascular structures and other organ systems. Additionally, a study demonstrated that approximately 25% of patients with pelvic ring disruption had lower urinary tract injuries. Recently, a systematic review revealed that approximately 29% of patients with pelvic fracture presented with genitourinary (bladder, urethra, and ureter) injuries [8]. These patients had potential urine contamination and urinary tract infections (UTIs). Furthermore, infectious complications, including pneumonia, UTI, surgical site infection, sepsis, and septic shock, have been noted in patients following pelvic fracture surgery [9]. However, there was little research about the mortality and readmission risk of the pelvic fracture patients when acquiring those complications.

A UTI was one of the common and early urinary complications with a pelvic fracture [10]. Surveillance and adequate treatment of UTIs were essential for patients with pelvic fractures for the following reasons: (1) for critical care of pelvic fracture patients in the intensive care unit, UTIs, and further, urosepsis and septic shock, all put patients at a higher risk of mortality [11]. Meanwhile, these patients may need prolonged urinary or suprapubic catheterization for the urinary tract injury from the pelvic. Moreover, repeated UTIs could increase the risk of genitourinary tract cancer [12]. (2) Stability of the biomechanical environment played a vital role in wound and fracture healing. Infection could affect the healing process by interrupting osteogenesis of osteoblast [13]. (3) A previous study showed that UTI increased the risk of longer hospitalization, more than 5 days in hip fracture [14]. However, there were no such data on the risk of adverse outcome of UTIs after pelvic fracture surgery in regards to the importance and necessity of these clinical circumstances.

The Poisson regression had residuals that were assumed to follow the Poisson distribution, when estimating the probability of a count of study events within a given time [15]. Moreover, the Cox regression and Kaplan–Meier analysis provided the hazard ratio and cumulative probability over follow-up time. Hung et al. estimated the risk of oral cancer in a high-risk individual and estimated the predictive model; they used Poisson regression analysis to calculate the incidence density ratio of oral cancer between groups, and a Cox proportional-hazards model was used to explore the risk factors of oral cancer incidence [16].

In the present study, we evaluated the risks of mortality and readmission due to a UTI within 30 days among patients with pelvic fractures. We hypothesized that patients with pelvic fractures accompanied by UTIs would have an increased risk of mortality and readmission.

## 2. Materials and Methods

### 2.1. Data Sources

We used data from the Longitudinal Health Insurance Database (LHID) 2000, a subset of the National Health Insurance Research Database, to evaluate the risk of mortality and the readmission rates among patients with or without UTIs following pelvic fracture surgery. The LHID2000 comprises the data of 1 million beneficiaries who insured by the National Health Insurance program in 2000. The research period of the present study was from 1 January 1997 to 31 December 2013 (17 years). This retrospective population-based cohort study was approved by the National Health Insurance Administration and the Institutional Review Board of Chung Shan Medical University (registration number: CSMUH CS16183).

### 2.2. Study Population

**Figure 1:**
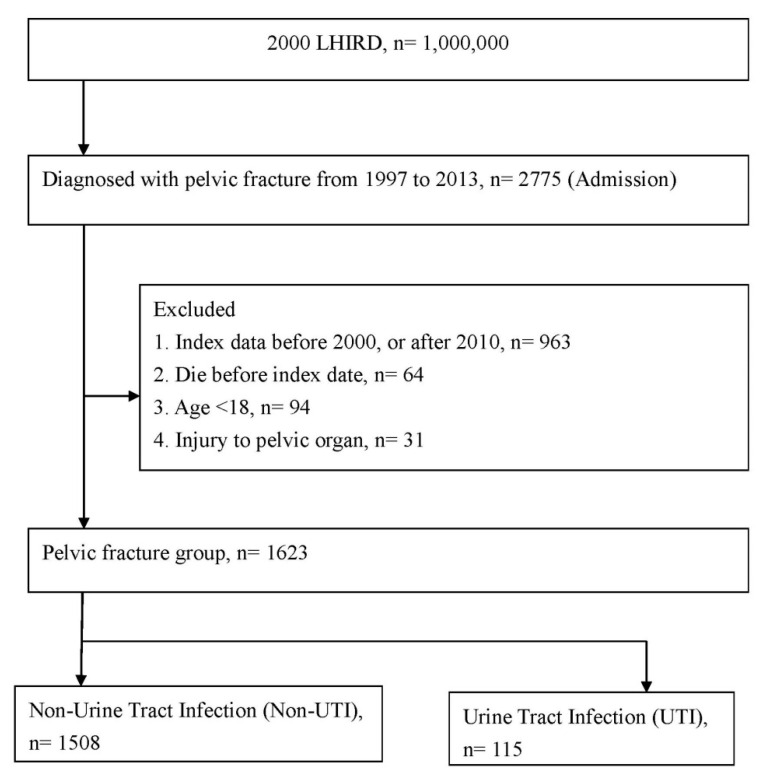
Flowchart for patient selection.

First, we included 2775 patients were hospitalized for pelvic fracture (as defined by the International Classification of Diseases, Ninth Revision, Clinical Modification [ICD-9-CM] code 808) between 1 January 1997 and 31 December 2013. The index date was 30 days after admission with a primary diagnosis of pelvic fracture. Second, we excluded patients who (a) had index dates before January 2001 (for left-censored or left-truncated data) or after December 2010 (limited due to the research timeframe); (b) died before the index date; (c) were aged <18 years, as the musculoskeletal conditions were not fully developed, which was a confounding factor; or (d) had injuries to pelvic organs, because this situation was a bias of mortality with readmission complications, and was not caused by pelvic fractures, but by pelvic organs injuries. Consequently, 1623 patients, including 115 patients with UTIs and 1508 patients without UTIs, were selected for analyses. Figure 1 illustrates the present study’s framework.

### 2.3. Characteristics, Comorbidities, and Study Outcomes

Baseline demographic characteristics, such as age and sex, were recorded. Comorbidities, including hypertension, diabetes mellitus, hyperlipidemia, coronary artery disease, previous incidence of a cerebrovascular accident, chronic kidney disease (CKD), COPD, chronic liver diseases, heart failure, depression, osteoporosis, asthma, and osteoarthritis within 1 year prior to the index date, were documented as potential confounding factors. The study outcomes were death and readmission. All included individuals received follow-ups from the index date to either the study event, the date of death, or the study’s conclusion (31 December 2013).

### 2.4. Statistical Analysis

Categorical data are presented as numbers and percentages; they were compared using a chi-squared test. The incidence rate with corresponding CIs and crude hazard ratios (HRs) were calculated using Poisson regression. After the proportional hazards assumption was tested, a Cox proportional hazards model analysis was performed to estimate the readmission rates, HRs for mortality, and 95% CIs. Statistical analysis was performed using SAS software version 9.4 (SAS Institute, Cary, NC, USA), and a *p*-value of <0.05 was considered statistically significant. The cumulative probabilities of mortality and readmission were assessed using a Kaplan–Meier analysis, in which statistical significance was determined by the results of a log-rank test.

## 3. Results

### 3.1. Characteristics of Study Patients

We identified 1623 patients who had been hospitalized for pelvic fracture from 1997 to 2013. Of these, including 115 with UTIs and 1508 without UTIs, were for analysis. Table 1 shows the baseline characteristics of the study participants. The proportion of female patients (73.91%) and patients over 65 years of age (45.22%) with UTIs was significantly higher than patients without UTIs. Compared with the non-UTI group, the UTI group had an older average age, and higher prevalence of comorbidities, such as hypertension, diabetes mellitus, hyperlipidemia, history of cerebrovascular accidents, chronic kidney disease (CKD), and heart failure, at baseline.

### 3.2. Risk of UTI after Exposure to Pelvic Fracture

We analyzed the incidence rates and HRs of mortality and hospital readmission among patients with pelvic fractures who did or did not have UTIs. The incidence rate of mortality among the UTI group increased at 3 months (adjusted HR: 2.50; 95% CI: 1.05–5.95), 6 months (adjusted HR: 2.31; 95% CI: 1.11–4.78), 9 months (adjusted HR: 2.71; 95% CI: 1.44–5.11), and 1 year (adjusted HR: 2.32; 95% CI: 1.25–4.29) after the index date. Additionally, the incidence rate of hospital readmission among the UTI group increased at 1 month (adjusted HR: 2.95; 95% CI: 1.71–5.10), 2 months (adjusted HR: 2.68; 95% CI: 1.73–4.15), 3 months (adjusted HR: 2.53; 95% CI: 1.71–3.73), 6 months (adjusted HR: 1.98; 95% CI: 1.40–2.80), 9 months (adjusted HR: 1.83; 95% CI: 1.33–2.51), and 1 year (adjusted HR: 1.72; 95% CI: 1.26–3.34) after the index date (Table 2).

**Figure 2:**
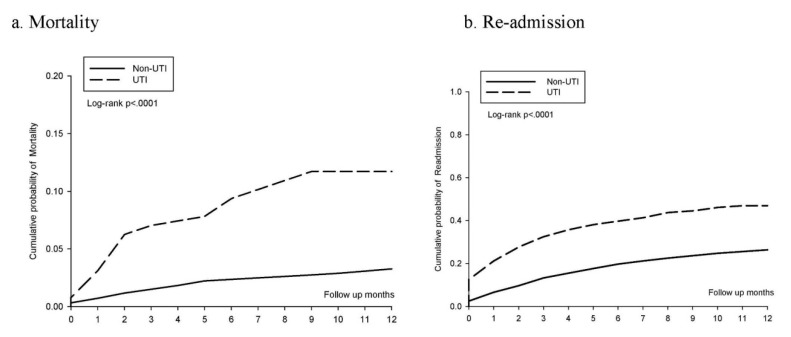
Kaplan–Meier curves of the cumulative proportions of mortality (**a**) and re-admission (**b**) in UTI and non-UTI.

We estimated the cumulative incidence of mortality and hospital readmission among patients in the UTI and non-UTI groups. The Kaplan–Meier curve for 1-year follow-up (Figure 2) indicated that those in the UTI group had an increased cumulative risk of both mortality (panel a, log-rank test *p* < 0.001) and hospital readmission (panel b, log-rank test *p* < 0.001) compared with the non-UTI group.

We used multiple Cox regression to estimate the hazard ratio (HR) for mortality and hospital readmission in the UTI group. The Cox regression was also used to explore the potential risk factors in the past study [17]. The results showed that the risk of both mortality (adjusted HR: 2.32; 95% CI: 1.25–4.29) and hospital readmission (adjusted HR: 1.72; 95% CI: 1.26–2.34) increased in the UTI group. Mortality and hospital readmission risks also increased in patients aged >65 years (Table 3). Patients with comorbidities, such as diabetes, previous cerebrovascular accidents, chronic kidney disease (CKD), chronic obstructive pulmonary disease (COPD) chronic liver diseases, osteoporosis, asthma, and osteoarthritis, had a higher risk of hospital readmission. Moreover, patients with CKD and heart failure had a higher mortality risk. Furthermore, patients who had received surgery for a UTI had a higher risk of hospital readmission (Table 3).

## 4. Discussion

We compared patients with pelvic fractures, with or without UTIs, to estimate the risk of mortality and hospital readmission. Our results demonstrated that risks of mortality and readmission increased in patients with pelvic fracture after 1 year if they had a UTI within 30 days of admission for pelvic fracture. The pelvic ring mainly consists of the sacrum, coccyx, pubis, ischium, and ilium. The pelvic cavity contains blood vessels, nerves, urogenital organs, and the rectum, which are important vitals in human. Hence, pelvic fracture is associated with a higher risk of injury to these internal organs and tissues [18]. In the present study, many injuries and complications occurred in patients with pelvic fractures after surgery. For example, unstable fractures were associated with complications such as retroperitoneal hematoma, injuries to the urethra or bladder, rectal injuries, and sciatic nerve damage [19]. This phenomenon might be related to postoperative medical care after discharge. By using national trauma data, Bjurlin et al. observed that 4.6% of patients with pelvic fractures had complications of genitourinary injuries [20]. Additionally, other complications, such as chest or wound infections and pain, might occur [21]. Due to the pain related to the injuries to their urinary systems, patients might exhibit difficulty passing urine. In this case, a urine catheter would always be required for urine drainage. Therefore, UTI risk may increase due to repeated catheterization [22].

In our study, we compared the incidence rates of both readmission and mortality among UTI and non-UTI patients after pelvic fracture. We found that the incidence density and adjusted hazard ratio (aHR) of readmission in the UTI group were higher than those in the non-UTI group, in each observed interval, from the index date to 1 year after the index date. Moreover, the incidence density and adjusted hazard ratio (aHR) of mortality in the UTI group were significantly higher than in the non-UTI group between the third month and 1 year after the index date. These results indicated that patients with pelvic fracture who experienced UTI complications postoperatively had a higher risk of both readmission and mortality up to 1 year after surgery. Malik et al. investigated the cause of readmission within 30 days due to adverse events among patients with pelvic/acetabular fractures. Malik et al. determined that many factors affected patient readmission, mortality, and other adverse events. For example, poor health status and concurrent comorbidities increased patients’ risks of adverse events after surgery. They also classified these adverse events into major (e.g., mortality) and minor ones (e.g., UTI) [9]. Dwyer and Moed observed that a small proportion of patients with a pelvic ring or acetabular fracture developed venous thromboembolism (VTE) within 90 days after hospital discharge [23]. The results of these studies allowed us to infer that different complications and adverse events could increase readmission and mortality rates in patients with pelvic fractures after surgery.

Poole and Ward observed that the overall mortality rate among approximately 500 patients who had an average age of 30 years, and average hospital stay of 16.5 days, was 8% [24]. Poole and Ward also noted that only 14.3% of deaths were directly associated with pelvic fracture. Most deaths were due to non-pelvic hemorrhage, multiple organ failure, and severe head injury [24]. In another retrospective study, in which 343 patients with pelvic fractures were enrolled, the overall mortality rate was 10.5% [25]. Chong et al. determined that one-third the related mortality rate was directly related to pelvic fracture, and another third of them was associated with complications [25]. In a national study of 31,380 patients with pelvic fractures, compared with patients without genitourinary injuries, those with such injuries were observed to have longer hospital stays, higher utilization of intensive care units, and an increased mortality rate (13.99% among patients with genitourinary injuries vs. 8.08% among patients without genitourinary injuries) [20].

Most studies have focused on the impact of readmission on hip fracture. Regardless of the different anatomical locations of both types of fractures, the available information on hip fracture in the literature might provide a key reference for those treating patients with pelvic fracture. Owing to the closely anatomical positions of hip and pelvic fractures, it was worth it to use relevant articles about hip fracture to explain our study. In fact, our study indicated that pelvic fracture patients complicated with UTI would have higher risk on mortality and readmission. The main cause of readmission among patients with hip fracture might be related to the development of certain medical complications after hospital discharge. Additionally, many predictors forecasted readmission among patients. A study in which patients with hip fractures were followed up for 1 year indicated that the associated readmission rate could be up to 21%; the main causes included bronchopneumonia, falls, urosepsis, cardiac exacerbations, and stroke after discharge [26]. Other predictors for readmission included pulmonary disease, deep vein thrombosis, heart failure, renal failure, sex, prolonged surgery time (i.e., >24 h), and preexisting diseases [27,28,29,30]. Moreover, fracture severity and advanced patient age were associated with higher mortality rates among patients with pelvic fractures [25]. Bjurlin et al. indicated that genitourinary injuries were not an independent factor behind mortality, but independent predictors were age of ≥65 years, initial systolic blood pressure measurements in the emergency department, Injury Severity Score, Glasgow Coma Scale, and female sex [20].

Some predictors of the mortality rates of patients with hip fracture after discharge have also been identified. Heyes’ group also studied the 1-year mortality rate among 465 patients with hip fracture. They determined the overall 1-year mortality rate to be 15.1%. Patients with a surgery time of ≥36 h had an increased risk of mortality within 1 year of surgery. Mortality risks were also associated with increased comorbidity, surgery type, independence following discharge, alcohol intake, history of smoking, readmission, and several biochemical markers [31]. More importantly, 1-year mortality among readmitted patients or patients with prolonged hospital stays was quite high [30]. Hence, our results indicated that UTIs might act as a mortality predictor among patients with pelvic fracture.

Some studies have reported that patients with pelvic fractures had high incidences of chronic pain and inhibited movement [32,33]. For this reason, we proposed that the patients hold their urine for a longer period of time. However, several reports have indicated that the prolonged holding of urine could allow for bacteria growth and increase the risk of a UTI [34,35,36]. Therefore, this factor may have increased the likelihood of such patients in developing a UTI. Moreover, our study results show that UTIs are common complications of pelvic fractures, and the mortality and rehospitalization rates of patients with UTIs are significantly higher than non-UTI patients. Therefore, more attention and aggressive treatments for these patients is our recommendation.

To enroll only patients with pelvic fractures, we excluded those who were diagnosed as having only an injury to the pelvis rather than pelvic fracture. However, some limitations in this study persisted. First, the database could not provide information regarding the severity and types of pelvic fractures in patients, such as ratings according to the Injury Severity Score and Glasgow Coma Scale. These scores might affect mortality and hospital readmission risks. Second, we did not know how much time passed between the pelvic fracture event and the time of surgery. However, delaying surgery might affect the prognosis of the fracture and increase the risk of readmission and mortality.

## 5. Conclusions

In conclusion, UTI could act as a risk factor and a predictor for patients with pelvic fractures. UTI complications experienced within 30 days could increase the risk of mortality and hospital readmission. To reduce the mortality of patients and to diminish medical burdens, physicians must pay more attention to postoperative care and monitor the conditions of these patients after discharge to prevent UTI complications.

## Figures and Tables

**Table 1:** Baseline characteristics among study groups.

Variables	Non-UTI *n* = 1508	UTI *n* = 115	*p*-Value
Sex			<0.0001
Female	823(54.58%)	85(73.91%)	
Male	685(45.42%)	30(26.09%)	
Age			<0.0001
18–40	551(36.54%)	31(26.96%)	
41–65	566(37.53%)	32(27.83%)	
>65	391(25.93%)	52(45.22%)	
Comorbidities			
Hypertension	358(23.74%)	52(45.22%)	<0.0001
Diabetes mellitus	216(14.32%)	31(26.96%)	0.0003
Hyperlipidemia	145(9.62%)	23(20%)	0.0004
Coronary artery disease	165(10.94%)	17(14.78%)	0.2083
Cerebrovascular accident	131(8.69%)	23(20%)	<0.0001
CKD	114(7.56%)	15(13.04%)	0.0361
COPD	169(11.21%)	16(13.91%)	0.3787
Chronic liver diseases	183(12.14%)	18(15.65%)	0.2698
Heart failure	72(4.77%)	14(12.17%)	0.0006
Depression	223(14.79%)	19(16.52%)	0.6148
Osteoporosis	233(15.45%)	25(21.74%)	0.0755
Asthma	75(4.97%)	9(7.83%)	0.1832
Osteoarthritis	378(25.07%)	37(32.17%)	0.0922
Surgery for pelvic fracture	177(11.74%)	11(9.57%)	0.4829

**Table 2:** Incidence rate for the mortality risk and re-admission.

Variable	Incidence Density * (95% CI)		cRR * (95% CI)	*p*-Value	aHR * (95% CI)	*p*-Value
	Non-UTI	UTI				
Mortality						
from index date to						
1 m	0.60(0.31–1.17)	2.67(0.86–8.30)	4.37(1.18–16.1)	0.0268	2.48(0.63–9.66)	0.1897
2 m	0.54(0.33–0.88)	2.25(0.93–5.41)	4.12(1.51–11.2)	0.0056	2.43(0.84–7.03)	0.1000
3 m	0.49(0.32–0.75)	2.44(1.22–4.89)	4.87(2.16–10.9)	0.0001	2.50(1.05–5.95)	0.0369
6 m	0.38(0.27–0.53)	1.72(0.95–3.12)	4.39(2.22–8.67)	<0.0001	2.31(1.11–4.78)	0.0241
9 m	0.31(0.22–0.42)	1.59(0.96–2.64)	5.02(2.78–9.07)	<0.0001	2.71(1.44–5.11)	0.0019
1 year	0.28(0.21–0.37)	1.19(0.71–1.98)	4.14(2.32–7.38)	<0.0001	2.32(1.25–4.29)	0.0070
Readmission						
from index date to						
1 m	4.93(3.90–6.22)	18.1(11.4–28.8)	3.47(2.07–5.83)	<0.0001	2.95(1.71–5.10)	0.0001
2 m	4.52(3.80–5.38)	14.5(9.95–21.1)	3.04(2.00–4.60)	<0.0001	2.68(1.73–4.15)	<0.0001
3 m	4.16(3.59–4.83)	12.3(8.78–17.3)	2.80(1.93–4.07)	<0.0001	2.53(1.71–3.73)	<0.0001
6 m	3.44(3.05–3.87)	8.17(6.00–11.1)	2.25(1.61–3.14)	<0.0001	1.98(1.40–2.80)	<0.0001
9 m	2.99(2.68–3.32)	6.61(4.95–8.83)	2.11(1.55–2.87)	<0.0001	1.83(1.33–2.51)	0.0002
1 year	2.61(2.37–2.89)	5.47(4.13–7.24)	1.98(1.47–2.67)	<0.0001	1.72(1.26–2.34)	0.0005

* Incidence rate, per 100 person months; cRR *, crude relative risk; aHR *, adjusted hazard ratio: adjusted for all variables.

**Table 3:** Multiple Cox regression to estimate the hazard ratio for the mortality risk and re-admission.

Variable	Mortality		Re-Admission	
	aHR (95% CI)	*p*-Value	aHR (95% CI) *	*p*-Value
UTI				
No	Reference		Reference	
Yes	2.32(1.25–4.29)	0.0070	1.72(1.26–2.34)	0.0005
Sex				
Female	Reference		Reference	
Male	1.19(0.68–2.09)	0.5248	1.23(1.01–1.51)	0.0382
Age				
18–40	Reference		Reference	
41–65	1.34(0.38–4.70)	0.6434	1.01(0.77–1.32)	0.9209
>65	6.55(2.03–21.0)	0.0016	1.57(1.14–2.14)	0.0047
Comorbidities (ref: non)				
Hypertension	1.16(0.63–2.15)	0.6229	0.99(0.76–1.28)	0.9528
Diabetes mellitus	1.05(0.58–1.91)	0.851	1.51(1.17–1.94)	0.0014
Hyperlipidemia	0.54(0.25–1.15)	0.1142	0.79(0.58–1.06)	0.1254
Coronary artery disease	0.55(0.29–1.02)	0.0581	0.88(0.66–1.18)	0.4048
Cerebrovascular accident	1.54(0.84–2.82)	0.1554	1.35(1.02–1.78)	0.0328
CKD	2.80(1.60–4.90)	0.0003	1.59(1.19–2.12)	0.0016
COPD	1.84(1.01–3.37)	0.0454	1.47(1.12–1.93)	0.0045
Chronic liver diseases	1.42(0.79–2.58)	0.2384	1.46(1.13–1.87)	0.0028
Heart failure	2.79(1.54–5.06)	0.0007	1.35(0.96–1.91)	0.0777
Depression	1.09(0.58–2.05)	0.7717	1.20(0.94–1.53)	0.1335
Osteoporosis	0.94(0.53–1.67)	0.8429	1.49(1.17–1.90)	0.0010
Asthma	1.51(0.76–3.00)	0.2349	1.46(1.01–2.09)	0.0411
Osteoarthritis	1.39(0.81–2.39)	0.2306	1.28(1.03–1.58)	0.0226
Surgery				
No	Reference		Reference	
Yes	0.58(0.13–2.50)	0.4691	1.82(1.38–2.40)	<0.0001

* aHR, Adjusted Hazard Ratio: Adjusted for all variables.

## Data Availability

Restrictions apply to the availability of these data. Data were obtained from the National Health Insurance database, and are available from the authors, with the permission of the National Health Insurance Administration of Taiwan.
